# Case report: novel *DNAH11* compound heterozygous variants including an exon 30–54 duplication in a child with a highly suggestive primary ciliary dyskinesia phenotype

**DOI:** 10.3389/fgene.2026.1857794

**Published:** 2026-06-04

**Authors:** Shun Wang, Yuyi Zhang, Min Wu, Yanyu He, Sainan Chen, Xueyun Xu, Meng Lv, Jiapeng Ji, Yuqing Wang

**Affiliations:** Department of Respiratory Medicine, Children’s Hospital of Soochow University, Suzhou, Jiangsu, China

**Keywords:** DNAH11 gene, exome sequencing, nasal nitric oxide, primary ciliary dyskinesia, qPCR

## Abstract

Primary ciliary dyskinesia (PCD) is a rare disorder characterized by dysfunction of motile cilia and chronic progressive respiratory disease, mianly inherited in an autosomal recessive manner. Biallelic variants in dynein axonemal heavy chain 11 (*DNAH11*) have been reported in association with PCD and are typically associated with normal ciliary ultrastructure. In addition, exon-level copy number variants (CNVs) in *DNAH11*, particularly duplications, remain poorly characterized and are prone to being overlooked by conventional sequencing workflows. We report a 9-year-old Chinese girl with recurrent lower respiratory tract infections, chronic pansinusitis, and bronchiectasis. Pulmonary function testing showed isolated small-airway dysfunction with preserved FEV1, with nasal nitric oxide (nNO) severely reduced (5.4 nL/min). Trio whole-exome sequencing integrated with read-depth CNV analysis detected novel compound heterozygous *DNAH11* variants: a paternal inherited missense variant (c.6556A>C, p.Thr2186Pro) and a previously unreported maternally derived intragenic duplication encompassing exons 30–54. The missense variant was confirmed by Sanger sequencing, and the exon-level dosage gain was confirmed by qPCR. This case expands the spectrum of *DNAH11* variants and highlights the importance of incorporating CNV detection into exome-based diagnostic process for children with a highly suggestive PCD phenotype.

## 1 Introduction

Primary ciliary dyskinesia (PCD, OMIM 244400) is a genetically heterogeneous disorder characterized by impaired function of motile cilia. It is most commonly inherited in an autosomal recessive manner, although autosomal dominant and X-linked forms have also been reported (Shoemark et al., 2025). Its prevalence is estimated to range from one in 10,000 to one in 40,000 individuals (Hunter-Schouela et al., 2023), and recent studies report a higher prevalence of approximately one in 7,600 (Hannah et al., 2022). PCD is clinically associated with recurrent respiratory infections, bronchiectasis, chronic sinusitis, otitis media with effusion, and infertility (Dong et al., 2022), approximately 50% of patients presenting with situs inversus (SI) at birth (Hunter-Schouela et al., 2023).

The diagnosis of PCD relies primarily on transmission electron microscopy (TEM), genetic testing, nasal nitric oxide (nNO) measurement, and high-speed video microscopy(HSVM) (Shoemark et al., 2025). Effective PCD management depends heavily on early diagnosis and intervention (Mirra et al., 2017; Shoemark et al., 2025). However, it is often delayed unless SI is detected, with most patients undergoing extensive evaluations before a definitive diagnosis (Kuehni et al., 2010; Lucas et al., 2020). In China, the diagnostic age in children ranges from 2 months to 14 years, which is significantly higher than in Western cohorts (Guan et al., 2021; Peng et al., 2022).

To date, more than 50 PCD-associated genes have been identified (Dong et al., 2025). Among these, dynein axonemal heavy chain 11 (*DNAH11*) variants are often associated with preserved axonemal ultrastructure, leading to normal TEM findings (Dougherty et al., 2016; Shapiro and Leigh, 2017). About 30% of PCD patients exhibit normal ciliary ultrastructure (Boon et al., 2014), and approximately 20% of these cases carry biallelic *DNAH11* variants (Knowles et al., 2012), resulting in a risk of missed diagnosis using conventional protocols (Shoemark et al., 2018). While single nucleotide variants (SNVs) in *DNAH11* are well-documented, exon-level copy number variants (CNVs), particularly duplications, remain poorly understood. Prior to this study, only one intragenic CNV (exon 64–65 deletion) has been reported with orthogonal validation (Dong et al., 2022). A large duplication (exon 7–14) was also recorded in a study, though it lacked orthogonal validation (Marshall et al., 2015). Notably, small in-frame duplications, such as a 21-bp duplication, have been observed but are typically classified as small in-frame duplications rather than CNVs (Ing et al., 2021). Comprehensive genetic testing can help define distinct phenotypic patterns and inform clinical evaluation and treatment (Horani and Ferkol, 2021).

We report a case of a 9-year-old Chinese girl with a highly suggestive PCD phenotype. Whole-exome sequencing (WES) identified novel compound heterozygous *DNAH11* variants: a paternal missense variant of uncertain significance (VUS) (c.6556A>C, p.Thr2186Pro), and a maternally inherited large intragenic duplication spanning exons 30–54, while previously reported *DNAH11* variants mainly comprise nonsense, missense, splice-site mutations, or small deletions (Xia et al., 2021). In this study, the duplication was characterized by segregation analysis, read-depth CNV analysis, *in silico* assessment, and quantitative PCR (qPCR). This case expands the mutational spectrum of *DNAH11* and supports the clinical utility of CNV analysis in exome testing for suspected PCD.

## 2 Case presentation

### 2.1 Ethical compliance and clinical examination

All procedures performed in this study were conducted in accordance with the Declaration of Helsinki (2013 revision). Informed consent was obtained from the patient’s parents, and ethical approval was obtained from the Institutional Ethics Board of Children’s Hospital of Soochow University (2024CS149). Routine blood tests, inflammatory markers, chest radiography, paranasal sinus and chest computed tomography (CT), pulmonary function testing (PFT), fractional exhaled nitric oxide (FeNO), and nNO measurement were performed as part of the initial clinical examination. Additional evaluations included bronchoscopy with bronchoalveolar lavage (BAL), and targeted next-generation sequencing (tNGS) of BAL fluid for pathogen detection.

### 2.2 Clinical overview

**FIGURE 1:**
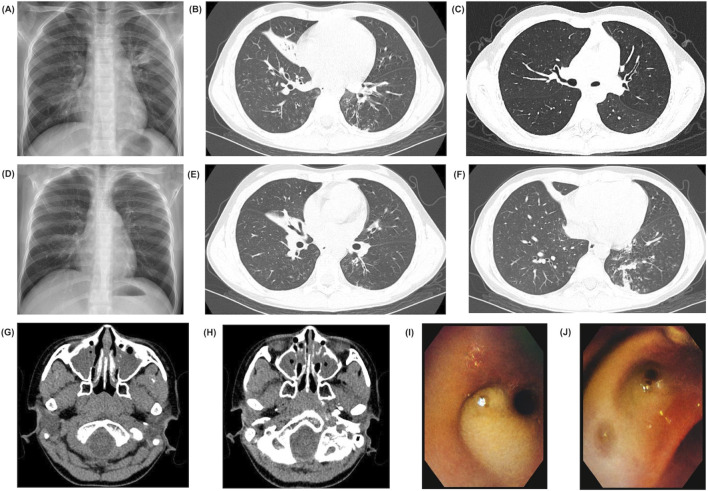
Radiological evolution and bronchoscopic findings of the proband. **(A–C)** Imaging obtained 5 months prior to the current admission. **(A)** Chest X-ray showing patchy opacities in the right lung. **(B,C)** Chest CT showing consolidation in the right middle lobe, with bronchiectasis in the left lung, indicating chronic airway damage. **(D–F)** Imaging upon current admission. **(D)** Chest X-ray with persistent high-density shadows in the right lower lung field. **(E,F)** Chest CT showing segmental consolidation, predominantly in the right middle lobe and left lingular segment. **(G,H)** Paranasal sinus CT: diffuse mucosal thickening and opacification of bilateral maxillary and ethmoid sinuses, confirming the diagnosis of pansinusitis. **(I,J)** Fiberoptic bronchoscopy: endoscopic view of the right middle and lower lobe bronchi showing mucosal hyperemia, edema, and obstruction of the lumen by copious purulent secretions.

A 9-year-old girl was admitted with a 5-day history of cough and intermittent fever. She had experienced a persistent wet cough and recurrent lower respiratory tract infections (LRTIs) since early childhood, as well as bronchopneumonia, eczema, wheezing, and allergic rhinitis. She had no history of otitis media, hearing loss, or neonatal respiratory distress. A chest X-ray obtained 5 months earlier demonstrated patchy high-density opacities in the right middle lobe and concomitant bronchiectasis in the left lung, which showed slow radiological resolution on regular follow-up (Figures 1A–C). She was born to non-consanguineous healthy parents, with no family history of recurrent respiratory infections, bronchiectasis, or infertility. Physical examination revealed bilateral coarse breath sounds with crackles in the right lung. Cardiac auscultation was unremarkable, consistent with situs solitus (SS).

### 2.3 Imaging and functional assessment

Chest radiography was performed using a digital radiography system (UHI-DR), paranasal sinus and chest CT were obtained with a GE CT scanner. Chest radiography and CT showed consolidation in the right middle lobe and left lingular segment (Figures 1D–F). Paranasal sinus CT demonstrated pansinusitis accompanied by mucosal thickening (Figures 1G,H).

PFT was performed using a Jaeger spirometer (Jaeger, Germany). Predicted values were calculated according to sex, age, and height, with the best of three technically acceptable maneuvers used for the final analysis. PFT revealed isolated small-airway dysfunction: forced vital capacity (FVC) and forced expiratory volume in 1 s (FEV1) were within the normal range, while forced expiratory flow at 50% and 75% of vital capacity (FEF50, FEF75) were markedly reduced, with a negative bronchodilator response. Full PFT parameters and results are presented in Supplementary Table 1.

FeNO and nNO were measured using an Aerocrine nitric oxide analyzer (Aerocrine AB, Solna, Sweden). FeNO was measured at a flow rate of 50 mL/s according to American Thoracic Society recommendations (Dweik et al., 2011), and nNO was assessed using the nasal aspiration method at 10 mL/s with the patient in the seated position(Shapiro et al., 2020). Both FeNO and nNO were tested during the patient’s acute respiratory infection episode at hospitalization. The measured FeNO value was 7 ppb, and the nNO concentration was 9 ppb, corresponding to a calculated production rate of 5.4 nL/min. The nNO measurement curve is provided in Supplementary Figure 1.

### 2.4 Bronchoscopy and microbiology

Sputum viral nucleic acid testing was positive for human rhinovirus, while sputum bacterial culture was negative. Fiberoptic bronchoscopy showed mucosal hyperemia and edema of the trachea and bilateral bronchi. The lumens of the right middle and lower lobes, together with the left lower lobe, were obstructed by copious thick purulent secretions (Figures 1I,J). The tNGS of BAL fluid identified *Haemophilus influenzae* (11,776 reads) and human rhinovirus (319 reads).

### 2.5 Treatment and outcome

**FIGURE 2:**
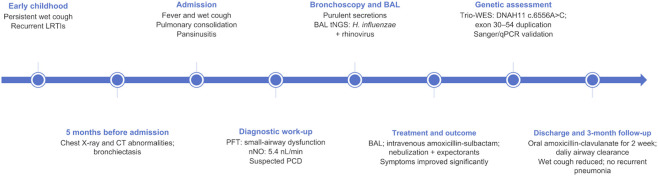
Timeline of the patient’s clinical course, diagnostic work-up, treatment, and follow-up.

On admission, the patient exhibited a poor response to initial oral cephalosporins therapy, with persistent high fever and wet cough. Fiberoptic bronchoscopy with BAL was then performed, and tNGS of BAL fluid identified *H. influenzae*. The antibiotic regimen was therefore switched to intravenous amoxicillin-sulbactam. Meanwhile, intensive airway clearance therapy was implemented, including nebulization medications and oral expectorants. The patient’s clinical symptoms improved markedly, and follow-up chest imaging demonstrated resolution of the pulmonary consolidation. After discharge, she completed a 2-week course of oral amoxicillin-clavulanate potassium granules, and was maintained on a PCD-oriented respiratory management plan, including daily airway clearance (inhaled beclomethasone dipropionate, compound ipratropium bromide, plus oral acetylcysteine granules) and symptomatic supportive care. At the 3-month follow-up, the frequency of wet cough was reduced markedly, with no recurrence of pneumonia, as outlined in the clinical timeline (Figure 2).

## 3 Diagnostic assessment

### 3.1 Genetic testing and data processing

Genomic DNA was extracted from peripheral blood samples obtained from the proband and her parents using a DNA extraction kit (CWE2100 Blood DNA kit V2; Beijing Kangwei Century Biotechnology Co., Ltd., Beijing, China) on a 96-channel automatic nucleic acid extraction machine from the same manufacturer. Sequencing was performed on a HiSeq 2,500 system (Illumina, San Diego, CA, USA). Data processing and bioinformatics analysis were performed using the Genome Analysis Toolkit (https://gatk.broadinstitute.org/hc/en-us).

Trio-WES was performed using paired-end sequencing after capture of coding exons and adjacent splice regions (±20 bp). Mean sequencing depth was 115.94× for the proband, 151.23× for the mother, and 143.50× for the father. Approximately 99.28% of the target region in the proband was covered at ≥20× depth (99.52% for the mother, 99.66% for the father), ensuring high resolution for single nucleotide variant (SNV) and CNV detection.

Population allele frequencies were queried in the Genome Aggregation Database (gnomAD v4.1.0; GRCh38, https://gnomad.broadinstitute.org/). Genomic coordinates are based on GRCh38/hg38. Gene nomenclature followed the HUGO Gene Nomenclature Committee (HGNC, http://www.genenames.org/) standards, and variants were annotated according to RefSeq (NM_001277115.2). Variant descriptions (at both nucleotide and amino acid levels) adhered to the Human Genome Variation Society (HGVS, http://varnomen.hgvs.org/) guidelines.

CNV calling was performed from exome read-depth data and visually inspected in Integrative Genomics Viewer (IGV, https://software.broadinstitute.org/software/igv/). For the exon 30–54 duplication, read depth across *DNAH11* was compared among the proband and both parents, with flanking exons serving as internal controls.

### 3.2 Sanger sequencing validation and qPCR dosage analysis

The *DNAH11* region encompassing NM_001277115.2:c.6556A>C was verified via Sanger sequencing. Capillary electrophoresis was performed on the ABI 3730 DNA Analyzer (Applied Biosystems). The primers used for Sanger were as follows: forward: 5′-CCC​TTC​TCG​ATC​TTA​GCA​CAC​ACA-3′; reverse: 5′-ACA​GGG​AGC​CCT​GAC​ATA​ATT​GAC-3′. Increased genomic dosage within the *DNAH11* exon 30–54 was confirmed using SYBR Green-based qPCR on a SLAN-96P real-time PCR system (Hongshi, China). Two assays targeting regions within the duplicated interval were analyzed (Assay 1: chr7:21658857–21659016; Assay 2: chr7:21744778–21745035), with normalization to a reference locus on chromosome 20 (chr20:37237635–37237740). Relative DNA dosage was interpreted relative to a diploid calibrator control (SD2), and the copy number values were treated as dosage estimates. The qPCR-validated interval was chr7:21658795–21750384, corresponding to approximately 91.6 kb. Because breakpoint-resolved sequencing was not performed, it represents an approximate validated region rather than the exact genomic size of the event.

### 3.3 Genetic analysis

**FIGURE 3:**
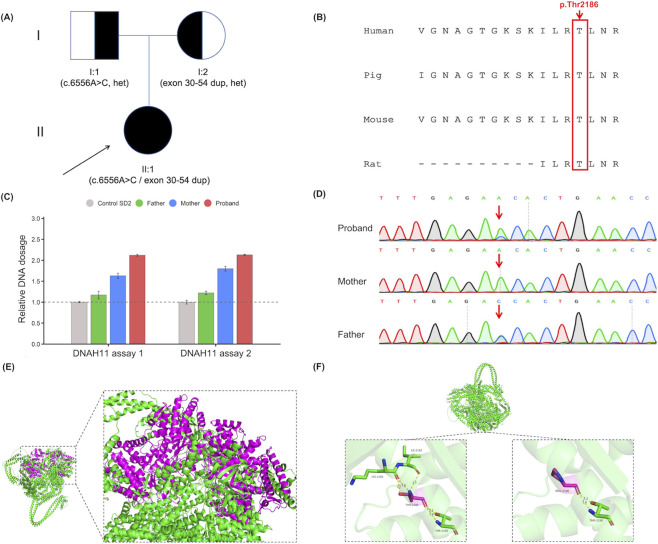
Genetic analysis and structural interpretation of the identified *DNAH11* variants. **(A)** Pedigree of the family with rare compound heterozygous *DNAH11* variants. The filled symbol (II:1) indicates the affected proband; half-filled symbols (I:1, I:2) indicate heterozygous carriers. The proband carries compound heterozygous variants: a paternal missense variant (c.6556A>C) and a maternal intragenic duplication involving exons 30–54. **(B)** Multiple sequence alignment of the *DNAH11* protein across different species. The red box indicates the highly conserved threonine residue at position 2,186 (p.Thr2186). **(C)** qPCR-based relative genomic DNA dosage analysis of the *DNAH11* exon 30–54 interval. Relative DNA dosage was normalized to the diploid control SD2. Two qPCR assays located within the predicted duplicated interval were tested: assay 1, chr7:21658857–21659016, and assay 2, chr7:21744778–21745035. The chr20:37237635–37237740 was used as the reference locus. Bars indicate mean ± SD. The proband and mother showed increased relative dosage at both assays, whereas the father showed no corresponding dosage gain. **(D)** Sanger chromatograms validating c.6556A>C in exon 40. Heterozygous A/C peaks are present in the proband and father but absent in the mother (indicated by red arrows). **(E)** AlphaFold-predicted structure of *DNAH11* showing the approximate structural context of the exon 30–54 duplication. The enlarged view indicates the motor region containing the AAA domains. This panel is shown for positional context only. **(F)** Local structural comparison of the p.Thr2186Pro variant based on AlphaFold and PyMOL. The wild-type and mutant models are shown side by side, with Thr2186 and Pro2186 highlighted in magenta, nearby residues shown as green sticks, and predicted local interactions indicated by dashed lines.

Trio-WES identified compound heterozygous variants in *DNAH11*, and the family pedigree and segregation pattern are summarized in Figure 3A. Cross-species alignment showed that Thr2186 is conserved among species (Figure 3B). A large intragenic duplication spanning exons 30–54 was suggested by read-depth analysis, IGV coverage inspection (Supplementary Figure 2) and exon-level BAM read-depth profiling (Supplementary Figure 3). qPCR analysis showed a dosage gain in the proband and mother relative to the diploid control, consistent with maternal inheritance of the duplication (Figure 3C). The missense variant NM_001277115.2:c.6556A>C (p.Thr2186Pro) was confirmed by Sanger sequencing, showing heterozygosity in the proband and father but not in the mother, consistent with paternal inheritance (Figure 3D).

Neither variant was observed in gnomAD v4.1.0 exomes or genomes (all and East Asian subsets). *In silico* predictions for c.6556A>C (p.Thr2186Pro) are summarized in Supplementary Table 2. SIFT (https://sift.jcvi.org), PolyPhen2 (http://genetics.bwh.harvard.edu/pph2/), and MutationTaster (http://www.mutationtaster.org) suggested a possible deleterious effect. MutationAssessor (http://mutationassessor.org/) was unavailable. LRT and REVEL scores were obtained from dbNSFP annotation data (http://www.dbnsfp.org/); LRT score was 0.003214, with LRT prediction of neutral, and REVEL was modest (0.375). SpliceAI (https://spliceailookup.broadinstitute.org/) predicted no splice-altering effect. Therefore, these computational tools data were considered only supportive.

Variant interpretation followed ACMG/AMP 2015 guidelines (Richards et al., 2015) for the SNV and ACMG/ClinGen 2020 constitutional CNV standards (Riggs et al., 2020) for the duplication. Both variants were formally classified as VUS. Although the patient had a highly suggestive PCD phenotype with markedly reduced nNO and biallelic *DNAH11* VUS findings, this case could not be classified as “comfirmed PCD” or “PCD highly likely” under the 2025 ERS/ATS framework because HSVM, immunofluorescence, and TEM were unavailable (Shoemark et al., 2025). Detailed ACMG-AMP and ClinGen CNV scoring criteria are provided in the Supplementary Material.

### 3.4 Structural and conservation analysis

To evaluate the potential functional impact of the identified variants, the *DNAH11* protein model (UniProt ID: Q96DT5) was retrieved from the AlphaFold Protein Structure Database (https://alphafold.ebi.ac.uk/). The exon 30–54 duplication maps to the motor region of *DNAH11* (Figure 3E). For the missense variant, substitution of threonine (Thr) by proline (Pro) may introduce conformational rigidity and alter local packing (Figure 3F). These structural observations suggest a possible local effect of p.Thr2186Pro, although functional validation is still needed.

## 4 Discussion

In this study, we identified a unreported large intragenic duplication spanning exons 30–54 of *DNAH11*, detected in trans with a rare missense variant in a child with highly suggestive PCD phenotype. This finding expands the spectrum of *DNAH11* variants and highlights the importance of CNV analysis in the evaluation of suspected PCD.

*DNAH11* encodes dynein axonemal heavy chain 11, a ciliary outer dynein arm heavy-chain motor protein. The gene is located on chromosome 7p15.3 and spans approximately 358,801 bp on GRCh38/hg38, with 82 annotated exons (https://www.ncbi.nlm.nih.gov/gene/8701). *DNAH11* is a microtubule-dependent motor ATPase involved in respiratory ciliary movement. *DNAH11*-related PCD can be diagnostically challenging because conventional TEM may be normal or non-diagnostic; therefore, integrated assessment using clinical features, nNO, genetic testing, and ciliary functional testing is important (Dougherty et al., 2016; Shoemark et al., 2025).

Because the exon-level duplication was inferred from read-depth analysis and qPCR dosage validation rather than breakpoint-resolved sequencing, its exact breakpoint configuration, orientation, and transcript-level consequence could not be determined. If the duplicated exons are arranged in tandem in the same orientation and are fully represented in the mature transcript, the event could theoretically alter the coding sequence. However, this remains a hypothetical scenario rather than a confirmed molecular consequence. On the other allele, structural modeling of p.Thr2186Pro suggests that substitution of threonine by proline at a conserved position may alter local packing and reduce compatibility with the local helical environment (Figure 3F).

Using standardized interpretation frameworks, we classified the paternal missense variant as a VUS in accordance with ACMG/AMP criteria, and the maternally inherited exon-level duplication conservatively as a VUS under the constitutional CNV standards. In this case, the combination of highly suggestive clinical features and biallelic *DNAH11* findings supports genotype-phenotype concordance, while the classification of each allele should still be interpreted cautiously given current evidence limitations.

A practical challenge in reported *DNAH11*-related PCD is that TEM can be non-diagnostic (Schultz et al., 2020). For these patients, the diagnosis often relies on integrated assessment of clinical phenotype, nNO, genetic findings, and ciliary functional studies (Pîrlog et al., 2025). In our case, the mean nNO level was 5.4 nL/min, which is far below the 2025 ERS/ATS diagnostic cutoff of <77 nL/min(Shoemark et al., 2025). Single-digit nNO values are extremely uncommon in secondary respiratory conditions (such as cystic fibrosis or acute viral sinusitis) that may cause transient nNO reduction (Lundberg and Weitzberg, 1999; Walker et al., 2012; Shapiro et al., 2018; Shoemark et al., 2025). Even though measured during an acute respiratory infection, the markedly reduced nNO in our study strengthened phenotype-genotype concordance, although TEM and HSVM would provide stronger diagnostic confirmation (Lucas et al., 2017; Shapiro et al., 2018).

PFT of the patient revealed isolated small-airway dysfunction, characterized by reduced FEF50 and FEF75 with preserved FVC and a negative bronchodilator response. This dissociation characterizes early-stage PCD lung disease (Kinghorn et al., 2020), due to structural remodeling and secretion retention of small airways(Marozkina, 2024). Reduced FEF25-75 often precedes FEV1 decline in PCD (Kinghorn et al., 2020; Shoemark et al., 2021). In this case, preserved FEV1 with a marked reduction in FEF75 (40% predicted) indicates that clinical monitoring in suspected or confirmed *DNAH11*-related PCD cases should incorporate sensitive small-airway indices. Moreover, although conventional cultures were negative, tNGS of BAL fluid detected *H. influenzae*, which may form biofilms hindering standard detection (Ademhan Tural et al., 2024; Bar-On et al., 2025).

Although *DNAH11* remains a leading cause of PCD in both Chinese and international cohorts (Zhang et al., 2025), published reports of *DNAH11* variants have been dominated by SNVs and small indels (Xia et al., 2021). To date, only three large intragenic CNVs in *DNAH11* have been reported in patients with PCD or suspected PCD phenotypes, as summarized in Table 1. The exon 64–65 deletion has been orthogonally validated by trio segregation analysis and RT-qPCR (Dong et al., 2022). In that prior case, the affected monozygotic twins presented with neonatal respiratory distress and discordant situs laterality (one with SI, the other with SS), a phenotype distinct from the patient in our study with SS and no history of neonatal respiratory distress. This divergence suggests that the location and type of CNV in *DNAH11* may influence the clinical features of PCD. Compared with this validated exon 64–65 deletion which spanned approximately 2.0 kb, the exon 30–54 duplication in our case covered a much larger qPCR-validated interval of approximately 91.6 kb.

**TABLE 1:** Comparison of reported large intragenic CNVs in *DNAH11*.

Characteristic	Case 1	Case 2	Case 3
Population	Chinese, 9-year-old female	Chinese, female monozygotic twins (diagnosed in neonatal period)	White, 6-year-old male
*DNAH11* variants (allele 1/allele 2)	Exon 30–54 duplication; c.6556A>C (p.Thr2186Pro)	Exon 64–65 deletion; c.2436C>G (p.Tyr812*)	Exon 7–14 duplication; c.10285C>A (p.Arg3429Ser)
CNV type	Duplication (25 exons)	Deletion (2 exons)	Duplication (8 exons)
CNV length	GRCh38/hg38 chr7:21658795–21750384; approximately 91.6 kb; qPCR-validated interval, breakpoint unresolved	GRCh38/hg38 chr7:g.21816397–21818402; approximately 2.0 kb	GRCh37/hg19 chr7: 21604615–21636907; approximately 32.3 kb; no orthogonal validation
Situs	SS	Discordant (one SI, 1 SS)	SI
nNO level (nL/min)	5.4	Not reported	4.9
Clinical phenotype	Recurrent LRTIs, chronic pansinusitis, small-airway dysfunction	Neonatal respiratory distress, atelectasis, recurrent cough	Not reported in detail
Key features/notes	Large intragenic *DNAH11* duplication identified; trio segregation confirmed in trans qPCR dosage validated; exon-level WES read-depth supported breakpoint and orientation unresolved	*DNAH11* deletion validated; trio segregation confirmed in trans; RT-qPCR validated; classified as LP	Normal TEM; overall unvalidated trio segregation not performed orthogonal validation not performed formal ACMG classification not assigned
References	Current study	Dong et al. (2022)	Marshall et al. (2015)

Abbreviations: CNV, copy number variant; SS, situs solitus (normal organ arrangement); SI, situs inversus; nNO, nasal nitric oxide; LRTIs, lower respiratory tract infections; LP, likely pathogenic; TEM, transmission electron microscopy; ACMG, american college of medical genetics and genomics.

The only other previously reported large *DNAH11* duplication, an exon 7–14 duplication, shares similarities with our case: both are large duplications identified in children with markedly reduced nNO (Marshall et al., 2015), the most specific non-invasive biomarker for PCD. However, this report lacked orthogonal validation of the duplication and trio-WES to confirm that the duplication was in trans with the paired missense variant. Our study reports a large intragenic *DNAH11* duplication with segregation analysis evidence and qPCR dosage validation, identified in a child with clinical symptoms suggesting PCD.

Beyond these large CNVs, a 21-bp in-frame microduplication (c.13523_13543dup21) in *DNAH11* has been reported (Ing et al., 2021). While not included in Table 1 due to its small size, this case suggests that *DNAH11* is susceptible to both large and small duplication. Notably, a somatic deletion involving *DNAH11* has been reported in a rare case of concurrent PCD and myelodysplastic syndrome secondary to chromothripsis, distinct from germline CNVs underlying inherited PCD (Agrawal et al., 2014).

A key limitation of this study is the lack of ciliary functional validation (due to the unavailability of fresh respiratory epithelial samples) and breakpoints analysis of the duplication. Therefore, the exact breakpoint, orientation, arrangement, and transcript-level consequence of the exon 30–54 duplication remain unresolved, and both variants remain VUS. The absence of ciliary functional validation prevented functional confirmation of PCD. However, trio segregation analysis, qPCR dosage validation, and the highly suggestive PCD phenotype provide supportive case-level evidence for the clinical relevance of the *DNAH11* findings.

## 5 Conclusion

In this study, we report a large intragenic duplication spanning exons 30–54 in *DNAH11*, identified in trans with a rare missense variant in a pediatric patient with a highly suggestive PCD phenotype. Both variants remain conservatively classified as VUS because ciliary functional evidence, transcript-level validation, and breakpoint sequencing were unavailable. This case expands the spectrum of *DNAH11* variants and highlights the importance of comprehensive genomic testing incorporating CNV calling, qPCR dosage validation, and segregation analysis in children with recurrent LRTIs and markedly reduced nNO.

## 6 Patient perspective

Before the clinical evaluation, we were concerned about her recurrent cough, nasal symptoms, and repeated lung infections, but we did not understand the underlying cause. The repeated clinic visits and treatments placed a burden on our family. After the clinical evaluation and treatment recommendations, we had a clearer understanding of her condition and of the importance of regular airway clearance and follow-up. Her symptoms improved after daily management, which reassured us. We agreed to share this case in the hope of improving awareness and earlier diagnosis of similar children.

## Data Availability

The original contributions presented in the study are included in the article/Supplementary Material, further inquiries can be directed to the corresponding author.
