# Supplementary material for: Case report: novel DNAH11 compound heterozygous variants including an exon 30–54 duplication in a child with a highly suggestive primary ciliary dyskinesia phenotype
Source: Front Genet. 2026 Jun 4;17:1857794. doi: 10.3389/fgene.2026.1857794 (PMC13275048; doi:10.3389/fgene.2026.1857794)
Supplement: Supplementary file 1 [file Supplementaryfile1.docx]

Supplementary Material

# Supplementary Figures and Tables

## Supplementary Tables

**Supplementary Table 1. Pulmonary function test results of the proband**

| **Parameter** | **Unit** | **Predicted** | **Measured (Best)** | **% Predicted** |
| --- | --- | --- | --- | --- |
| **Spirometry** |  |  |  |  |
| FVC | L | 2.06 | 2.00 | 97.4 |
| FEV1 | L | 1.77 | 1.58 | 89.6 |
| FEV1/FVC | % | 84.95 | 79.1 | 93.1 |
| **Small-airway Function** |  |  |  |  |
| PEF | L/s | 4.25 | 3.12 | 73.5 |
| FEF25 | L/s | 3.83 | 2.92 | 76.2 |
| FEF50 | L/s | 2.70 | 1.57 | **58.3** |
| FEF75 | L/s | 1.38 | 0.55 | **40.0** |
| MMEF 75/25 | L/s | 2.35 | 1.40 | **59.6** |
| **Bronchodilator Test** |  |  |  |  |
| ΔFEV1 | % | - | 1.5 | - |
| Conclusion |  |  | Negative |  |

*Abbreviations: FVC, forced vital capacity; FEV1, forced expiratory volume in 1 second; PEF, peak expiratory flow; MMEF, maximal mid-expiratory flow; FEF, forced expiratory flow; FEF25, FEF50, and FEF75 correspond to instantaneous flows at 25%, 50%, and 75% of the FVC exhaled, respectively; ΔFEV1, change in forced expiratory volume in 1 second after bronchodilator administration. Note: Values in bold indicate results significantly reduced (<65% predicted), suggesting small-airway dysfunction.*

**Supplementary Table 2. Population frequency and *in silico* prediction results for *DNAH11* NM_001277115.2:c.6556A>C (p.Thr2186Pro).**

| Database / tool | Score / result | Reference score | Interpretation |
| --- | --- | --- | --- |
| dbSNP rsID | NA | - | No rsID available |
| gnomAD v4.1 exome, all | Not observed | Absence supports rarity | Absent |
| gnomAD v4.1 exome, East Asian | Not observed | Absence supports rarity | Absent |
| gnomAD v4.1 genome, all | Not observed | Absence supports rarity | Absent |
| gnomAD v4.1 genome, East Asian | Not observed | Absence supports rarity | Absent |
| SIFT | 0.002, D | <0.05 = deleterious; ≥0.05 = tolerated | Deleterious |
| PolyPhen-2 HDIV | 1.000, D | D = probably damaging; P = possibly damaging; B = benign | Probably damaging |
| PolyPhen-2 HVAR | 0.987, D | D = probably damaging; P = possibly damaging; B = benign | Probably damaging |
| LRT | 0.003214, N | D = deleterious; N = neutral; U = unknown | Neutral |
| MutationTaster | 0.999133, D | D = disease-causing; P = polymorphism | Disease-causing |
| MutationAssessor | NA | High / medium / low / neutral functional impact if available | Not available |
| REVEL | 0.375 | Higher scores indicate stronger predicted pathogenicity | Modest |
| SpliceAI acceptor loss | 0 | ≥0.2 suggests possible splice effect; ≥0.5 provides stronger support | No predicted effect |
| SpliceAI donor loss | 0 | ≥0.2 suggests possible splice effect; ≥0.5 provides stronger support | No predicted effect |
| SpliceAI acceptor gain | 0 | ≥0.2 suggests possible splice effect; ≥0.5 provides stronger support | No predicted effect |
| SpliceAI donor gain | 0 | ≥0.2 suggests possible splice effect; ≥0.5 provides stronger support | No predicted effect |

*Abbreviations: NA, not available.*

## Supplementary Figures


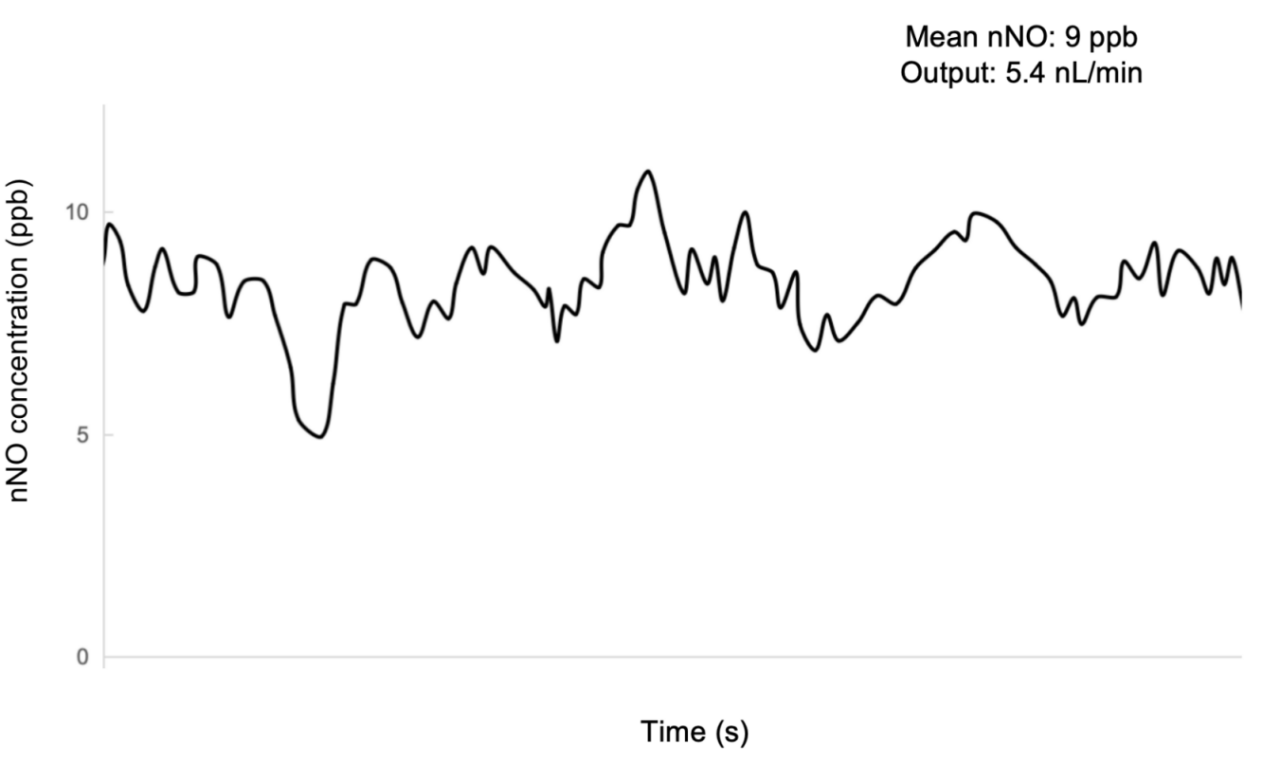


**Supplementary Figure 1.** Nitric Oxide Measurement.

The panel shows nasal nitric oxide (nNO) measured during hospitalization at a flow rate of 10 mL/s, during an episode of acute respiratory infection. The tracing showed a low signal with a recorded nNO value of 9 ppb, corresponding to 5.4 nL/min, markedly below the commonly used diagnostic standard for PCD.


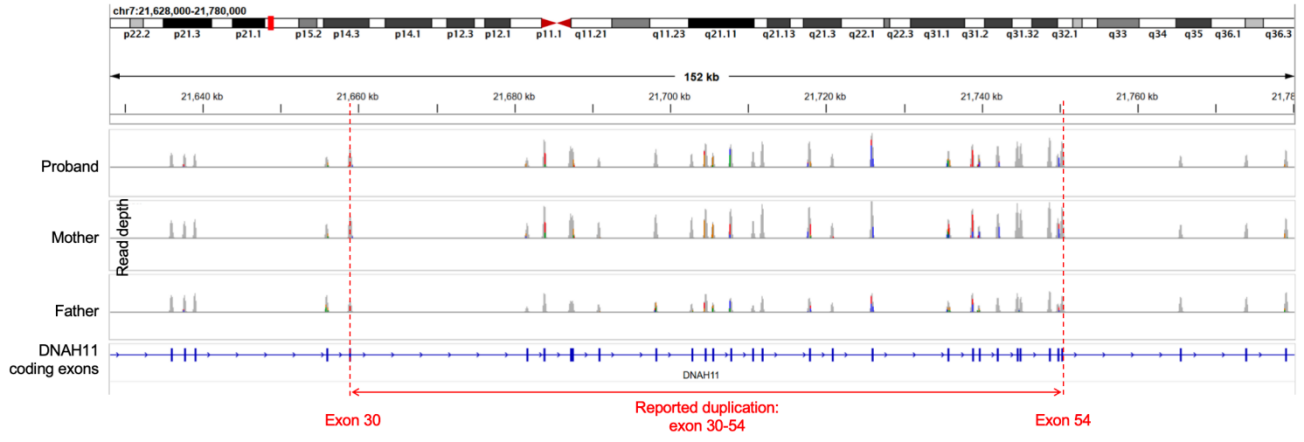


**Supplementary Figure 2.** Integrative Genomics Viewer (IGV) coverage plot demonstrating the copy number variant (CNV).

The red dashed vertical lines indicate the reported exon 30–54 duplication interval. The proband and mother show increased read depth across the marked interval compared with the father, while flanking regions outside the interval show no consistent dosage gain.


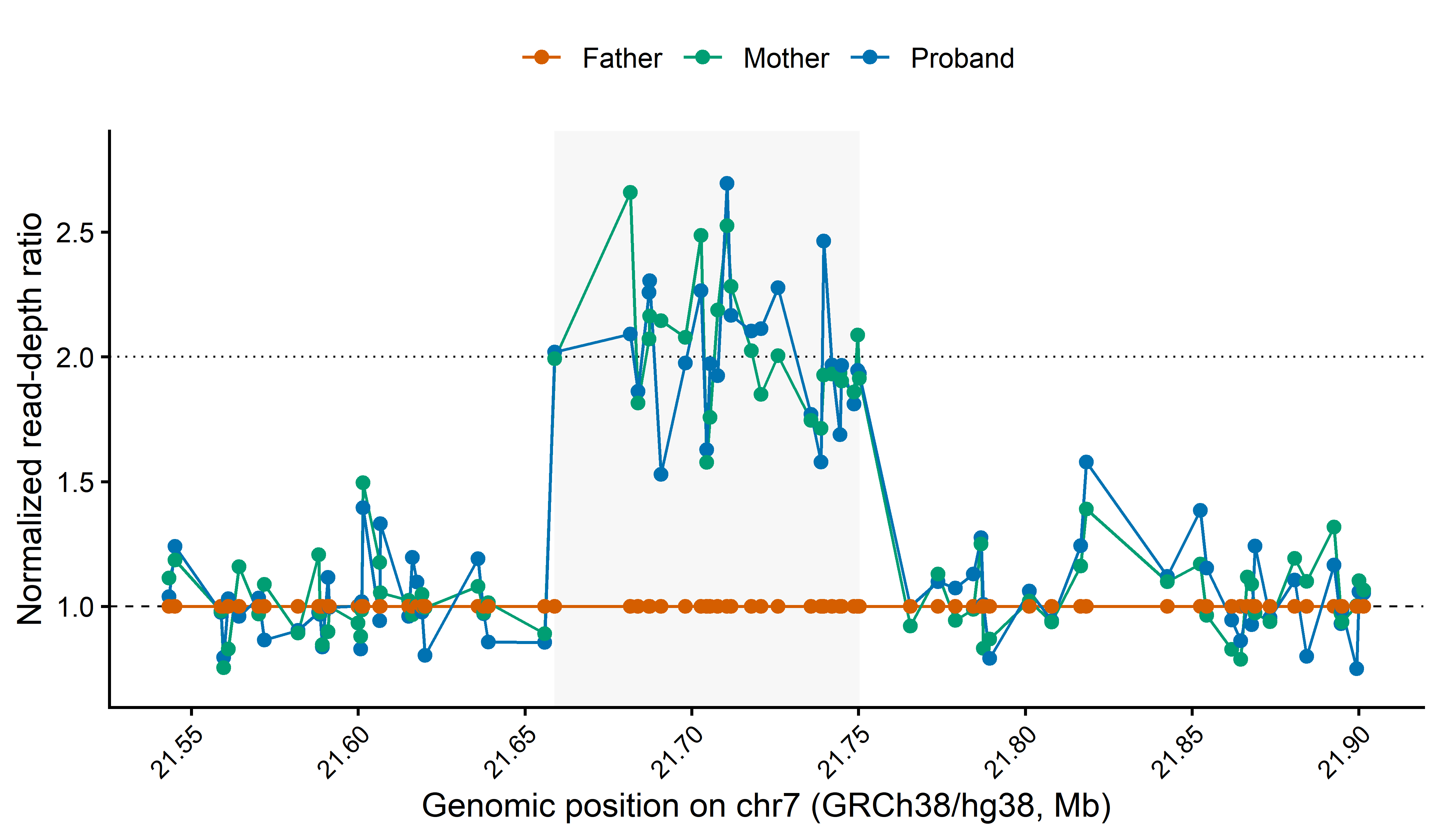


**Supplementary Figure 3.** Exon-level WES read-depth profile across *DNAH11* in the trio.

Normalized exon-level read-depth ratios across *DNAH11* are shown for the father, mother, and proband. Ratios were normalized to the unaffected father after correction using captured exonic regions outside the reported duplicated interval; the father is shown as the reference baseline. The shaded region indicates the reported duplicated interval, chr7:21658795–21750384 (GRCh38/hg38), corresponding to *DNAH11* exon 30–54 duplication supported by genomic DNA qPCR dosage validation. The proband and mother showed increased read-depth ratios within the reported duplicated interval. Exonic regions outside the reported interval remained overall near baseline and did not show a consistent dosage increase.

# Variant Classification

# **2.1 Missense variant (c.6556A>C, p.Thr2186Pro)**

]Classified as a Variant of Uncertain Significance (VUS) per ACMG-AMP 2015 guidelines:

PM2_Supporting: Absent from gnomAD v4.1.0, 1000 Genomes and ChinaMAP databases.

PP3: Not applied. Although several tools suggested a possible deleterious effect, the overall computational evidence was not fully concordant and the REVEL score was modest. Therefore, it was insufficient for PP3.

PP4: Not applied. Although the patient showed a highly suggestive PCD phenotype, ciliary functional confirmation was unavailable, and the phenotype was not used to upgrade the missense variant classification.

**2.2 Exon 30**–**54 duplication**

Classified as a Variant of Uncertain Significance (VUS) per 2020 ACMG/ClinGen CNV standards, with a total score of 0.45 (VUS range: -0.89 to 0.89):

+0.45 points (Section 4B): This intragenic duplication segregates in trans with a rare missense variant (c.6556A>C) in a patient with a highly suggestive PCD phenotype; the duplication spans the core AAA motor domains critical for dynein protein function, further supporting its potential clinical relevance.

0 points: *DNAH11* is an autosomal recessive disease gene with no established triplosensitivity or gain-of-function mechanism (ClinGen Haploinsufficiency Score=0, Triplosensitivity Score=0). No definitive transcriptomic or functional evidence confirms the duplication causes loss of gene function, thus Section 3 loss-of-function evidence criteria are not applied for this variant.

While both variants are formally classified as VUS, their in trans segregation, qPCR dosage validation, exon-level WES read-depth analysis, and the patient’s highly suggestive PCD phenotype provide supportive case-level evidence for the possible clinical relevance of the *DNAH11* findings.
